# Identification of disulfidptosis in esophageal squamous cell carcinoma based on single-cell and bulk RNA-seq data to predict prognosis and treatment response

**DOI:** 10.3389/fimmu.2025.1567793

**Published:** 2025-04-15

**Authors:** Xiaodan Zhang, Jianting Du, Xiao Lin, Shuliang Zhang, Taidui Zeng, Maohui Chen, Guanglei Huang, Chun Chen, Bin Zheng

**Affiliations:** ^1^ Department of Thoracic Surgery, Fujian Medical University Union Hospital, Fuzhou, Fujian, China; ^2^ Key Laboratory of Cardio-Thoracic Surgery(Fujian Medical University), Fujian Province University, Fuzhou, Fujian, China

**Keywords:** disulfidptosis, esophageal squamous cell carcinoma, prognostic analysis, single-cell transcriptomic analysis, therapeutic strategy

## Abstract

**Purpose:**

Our study aims to identify the molecular subtypes of genes associated with disulfidptosis in esophageal squamous cell carcinoma(ESCC), develop a prognostic model, and identify potential therapeutic targets.

**Methods:**

Based on the GSE53625 expression profile data, we identified molecular subtypes with significant survival differences through consensus cluster analysis. Subsequently, univariate Cox, multivariate Cox, and LASSO-Cox regression analysis were used to establish risk stratification models. The transcriptome data of the TCGA-ESCC cohort and the GSE160269 single-cell sequencing dataset were integrated to verify the biological significance of the model, and further analyze the heterogeneity of the tumor immune microenvironment, explore the differences in the intercellular communication network, and screen potential targeted drugs, providing a theoretical basis for subsequent translational research.

**Results:**

We identified two distinct patterns of disulfidptosis expression with significant differences in overall survival. Then, we constructed the prognostic signature of disulfidptosis, and results showed patients with high score had worse prognosis. Univariate and multivariate Cox analysis demonstrated that the constructed prognostic signature was an independent prognostic factor and was validated in an independent validation set. The two subgroups differed in the proportion of immune cell infiltration and related signaling pathways in ESCC. The exploration of immunotherapy data confirmed our prognostic signature also had certain predictive power for immunotherapy. Drug screening suggested AZD8186 and JQ1 as potential therapies for high-score patients.

**Conclusion:**

This study provides a new prognostic signature for ESCC, explores new therapeutic targets, and provides new theoretical support for personalized treatment.

## Introduction

Esophageal cancer (EC) is one of the world’s deadliest cancers, consisting primarily of esophageal squamous cell carcinoma (ESCC) and esophageal adenocarcinoma (1). Among them, ESCC is a highly lethal cancer in the esophagus, primarily concentrated in Asia and Africa (2, 3). Currently, the primary clinical treatments for ESCC are surgical resection, radiotherapy, and chemotherapy. However, most patients are already in the middle to late stage when detected because the early symptoms of ESCC are not obvious, leading to unsatisfactory treatment outcomes (4). Additionally, one of the core features of cancer is the ability to escape cell death, posing a great challenge for oncology treatment (5). Therefore, developing reliable and effective biomarkers and guiding individualized and optimal treatment modalities for ESCC patients is important.

Previous studies discovered that cancer cells can autonomously alter the flux of various metabolic pathways to increase bioenergy and biosynthetic demands and to mitigate the oxidative stress required for cancer cell proliferation and survival (6). When the metabolite accumulation exceeds the metabolic load of tumor cells, excess oxidative stress is generated, resulting in cell death (7). A recent study has identified a previously unexplained cell death caused by the rapid accumulation of excess cystine intracellular due to disulfide stress (8). This study discovered that in glucose-deprived SLC7A11-high cancer cells, intracellular NADPH is rapidly depleted (8, 9). The massive accumulation of disulfide molecules leads to abnormal disulfide bond cross-linking between actin cytoskeletal proteins and cytoskeletal contraction, disrupting their organization and resulting in actin network collapse and cell death, possibly leading to a new form of cell death, namely disulfidptosis (8, 10). Additionally, glucose transporter protein inhibitors could effectively inhibit cellular glucose uptake, causing NADPH depletion, actin cytoskeleton cross-linking, and disulfidptosis in SLC7A11-high cancer cells (10, 11). *In vivo* experiments on mice also demonstrated that GLUT inhibitors significantly inhibited tumor growth and induced aberrant disulfide cross-linking of actin cytoskeletal proteins in SLC7A11-high (10). This finding promises to be a new area of tumor therapy, but further research and exploration are needed to understand its specific mechanisms and therapeutic applications.

Currently, the study of disulfidptosis is still in its infancy, but relevant data are available to present its huge relationship with tumor development, such as lung and bladder cancers (12, 13). Similarly, we performed an analysis based on ESCC to explore the disulfidptosis-related genes expressions in specific carcinomas, resolve possible molecular linkages, and investigate potential prognostic targets. Therefore, this study aims to identify the molecular isoforms of disulfidptosis-related genes in ESCC in public databases, resolve the regulation pattern of disulfidptosis in ESCC, and construct a scoring model for disulfidptosis-related genes. Single-cell RNA sequencing (scRNA-seq) and bulk RNA-seq data were combined to explore the different tumor growth patterns, clinical outcomes, immune microenvironment, and cellular communication and predict new potential compounds to provide new theoretical support for clinical decision-making.

## Materials and methods

### Data collection and preprocessing

We obtained the GSE53625 dataset (gene expression profiles and clinical data) from the Gene Expression Omnibus (GEO). After filtering, 179 tumor samples with complete expression and survival (overall survival [OS]) data were included as the training cohort. The R package TCGAbiolinks was used to download FPKM expression profiles (for log transformation), survival data, and clinical data from the TCGA-ESCA EC and kept 80 squamous cancer samples with both expression and survival data for validation. The expression profiles (UMI-count) of 60 squamous cancer samples from the GSE160269 single-cell dataset were selected for analysis in this project. A dataset of cutaneous melanomas treated with anti-CTLA-4 was downloaded and used to assess the predictive efficacy of signature in the immunotherapy cohort (14). Interaction relationships of disulfidptosis-related genes were obtained based on the Search Tool for the Retrieval of Interacting Genes/Proteins (STRING) database (https://www.string-db.org/), and Protein-Protein Interaction Networks (PPI) were constructed based on the interactions (10).

### Consensus unsupervised clustering

Based on the expression profile data of 10 disulfidptosis-related genes, unsupervised cluster analysis was applied to identify different expression patterns of disulfidptosis. The ConsensusClusterPlus package was used for the operation, the distance used for clustering was Pearson, the clustering method was Pam, and 1000 replications were performed to ensure the stability of the classification. The R packages survminer and survival were used to generate survival curves for prognostic analysis using the Kaplan-Meier method. The log-rank test was used to determine the significance of differences and resolve the correlation between samples with different expression patterns and OS. Additionally, the R package limma was used to identify differentially expressed genes between different subgroups. We identified differentially expressed genes with |log2FC| ≥ 0.585 (fold change ≥1.5) and FDR < 0.05.

### Constructing a prognostic signature

The hazard ratio (HR) and prognostic significance of differential genes and screened genes with p < 0.05 as prognostic correlates were determined using univariate Cox regression analysis.

We applied LASSO regression (glmnet R package) to select key prognostic genes. The risk score was calculated by summing the product of each gene’s expression level (exp) and its LASSO coefficient (coef): Score = **Σ**exp*coef. The samples were divided into high and low groups according to the score. We generated Kaplan-Meier survival curves to compare patient outcomes. The significance of the differences was determined using the log-rank test to further resolve the correlation between these two types of samples and OS. The predictions of the scoring system on scoring were evaluated using the receiver operating characteristic curve (ROC), and the area under the curve (AUC) was visualized using the R package timeROC. Moreover, univariate and multivariate Cox analysis were performed to explore the independent prognostic value of the score.

### Gene set variation analysis and functional enrichment

We performed Gene Ontology (GO) and Kyoto Encyclopedia of Genes and Genomes (KEGG) pathway analyses using the clusterProfiler R package (parameters pvalueCutoff = 0.05, pAdjustMethod = “BH”), and the R package GSVA was used to perform genes differentially expressed in the two groups of samples were screened using the difference multiplicity |log2FC| ≥ 0.585 (difference multiplicity great ne set variation analysis to annotate the potential functions of key genes and predict their potential molecular functions. Gene sets were downloaded from the Molecular Signatures Database (MSigDB) for the HALLMARK, KEGG, and Gene Ontology Biological Processes (GOBP) sublibraries and used to conduct GSVA analysis.

### Tumor immune microenvironment assessment

The immune score, stromal score, and tumor purity were calculated for each tumor sample using the ESTIMATE algorithm. The Spearman correlation was calculated between score and immune score, stromal score, and tumor purity. According to previous studies, the single-sample gene-set enrichment analysis (ssGSEA) algorithm was used to estimate the relative abundance of each cell infiltrate in TME. The distribution of immune cell infiltrates were compared between different grouped samples using the Wilcoxon test.

### Predicting drug sensitivity

Based on the Genomics of Drug Sensitivity in Cancer (GDSC) (https://www.cancerrxgene.org/) and Chemical and Systems Biology Program (CTRP) (https://portals.broadinstitute.org/ctrp/) cancer genomics drug susceptibility databases, R package oncoPredict’s calcPhenotype algorithm was used to evaluate the drug IC_50_ values for each sample in the training set. The spearman correlation was calculated between score and drug IC_50_ to assess the correlation between drug sensitivity and signature. The difference between drugs IC_50_ in high and low groups was compared.

### Single-cell transcriptome data quality control and identification of malignant cells

After the original authors’ quality control, 60 ESCC samples were analyzed from the GSE160269 dataset using the R package Seurat (v4.1.0) and normalized using the NormalizeData function. The FindVariableFeatures function was used to identify highly variable genes based on their average expression values (greater than 0.1 and less than 8) and dispersion (greater than 1) to identify highly variable genes. Batch correction between samples was performed using the R package Harmony to avoid batch effects interfering with downstream analysis. Then, the data were scale-transformed, principal component analysis was used for dimensionality reduction, the top 50 principal components were selected for downstream analysis, and the RunUMAP function was used for visualization.

The cell type annotation information provided by the original authors of the dataset was extracted. Among them, the malignant epithelial cells were identified using the copykat method. The CellScore was calculated using the AddModuleScore function of the Seurat package based on the signature-containing genes. Malignant epithelial cells were classified into high and low groups based on the median CellScore of malignant epithelial cells. Cell stemness scores were calculated using the AddModuleScore function and stemness gene sets from previously published studies.

### Trajectory analysis and cellular communication

The R package monocle2 was used for trajectory analysis, time series analysis of malignant epithelial cells was proposed, and the R package CellChat was used for cell-to-cell communication analysis.

### Statistical analysis

All analyses were performed using R version 4.1.2. For significance analysis between various values (expression, infiltration ratio, and various eigenvalues), the Wilcoxon rank sum test was used to compare differences between two groups of samples, whereas the Kruskal-Wallis was used to compare differences between multiple groups of samples. For plot presentation, ns indicates p > 0.05, * indicates p < 0.05, ** indicates p < 0.01, *** indicates p < 0.001, and **** indicates p < 0.0001. Survival curves were generated for the prognostic analysis by the Kaplan-Meier method, and determined the significance of differences using the log-rank test.

## Results

### Consensus clustering identifies sample subgroups

**Figure 1:**
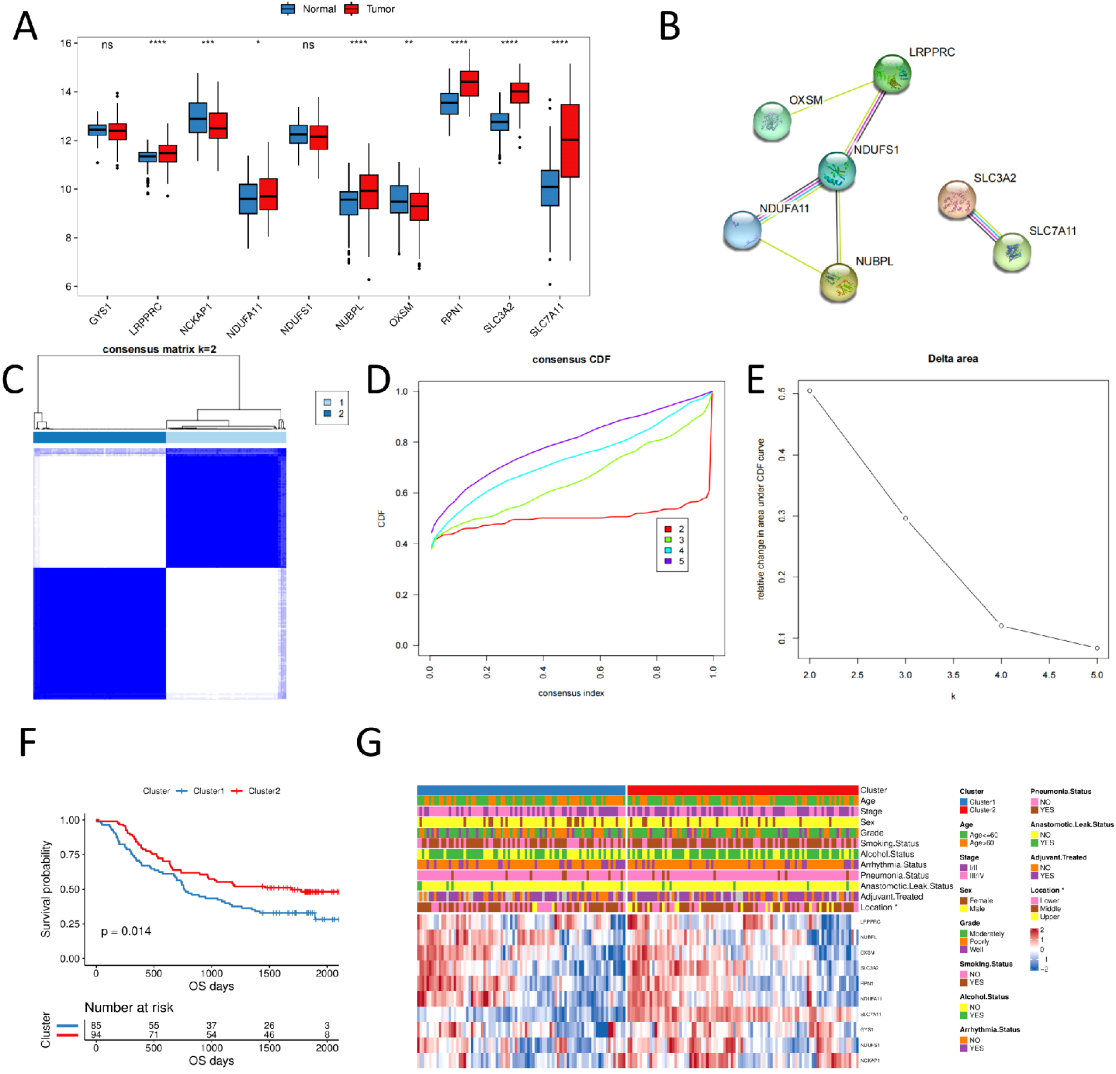
Consensus clustering of disulfidptosis-related genes in ESCC samples **(A)** expression of disulfidptosis-related genes in normal and tumor samples; **(B)** PPI of disulfidptosis-related genes; **(C)** consensus clustering of disulfidptosis-related genes, 1 and 2 represent two subgroups; **(D)** consensus clustering CDF plot; **(E)** consensus clustering cumulative distribution function Delta area; **(F)** OS curves of two subgroups; **(G)** clinicopathological characteristics and expression level heat plot of disulfidptosis-related genes in two subgroups. (ns, p > 0.05; *, p < 0.05; **, p < 0.01; ***, p < 0.001; ****, p < 0.0001).

We compared their expression differences between normal and tumor samples to assess whether the expression of disulfidptosis-related genes affects tumor progression in ESCC. The results revealed that six genes were significantly highly expressed in tumor samples, whereas two genes were significantly low (**Figure 1A**). NDUFS1 showed the highest connectivity in the protein interaction network (**Figure 1B**), suggesting its potential as a hub gene regulating disulfidptosis.

We performed unsupervised cluster analysis using the R package ConsensusClusterPlus based on 10 disulfidptosis-related genes expression profiles of ESCC samples from the GSE53625 dataset. We identified two subgroups, named Cluster1 and Cluster2 (N = 85/94, **Figures 1C–E**). The Kaplan-Meier curves revealed that the two subgroups had significantly different prognoses, with Cluster1 having a worse OS (**Figure 1F**). We also compared the clinicopathological characteristics of different subgroups of ESCC using a heat plot. We discovered significant differences in the distribution of esophageal carcinoma sites (locations) between subgroups of patients (p < 0.05, **Figure 1G**).

### Construction and validation of the prognostic signature

**Figure 2:**
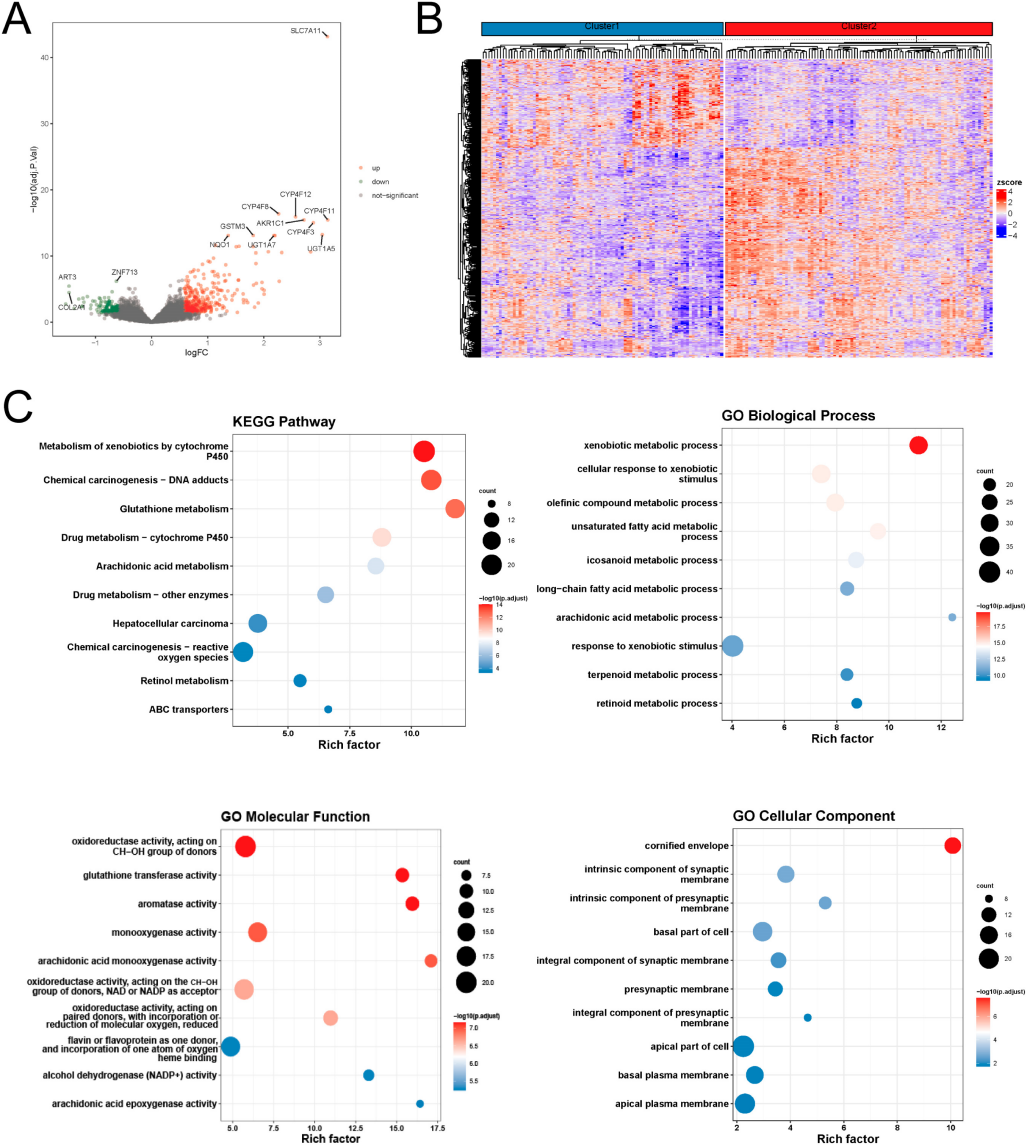
Molecular differences in disulfidptosis expression patterns **(A)** differential volcano plot; **(B)** differential gene expression heat plot; **(C)** functional enrichment analysis of differential genes.

We constructed a disulfidptosis signature for predicting the prognosis of ESCC patients based on differential genes between the disulfidptosis expression patterns. Initially, we screened 493 differentially expressed genes between the two disulfidptosis expression patterns using the limma package (**Figures 2A, B**). GO and KEGG enrichment analysis of these differentially expressed genes using the clusterProfiler package. The KEGG results indicated that these genes were primarily enriched in pathways related to cytochrome P450, DNA adducts, reactive oxygen species (ROS), suggesting potential associations with drug toxicity, drug resistance, or metabolism of environmental carcinogens, involving mechanisms of carcinogenesis driven by DNA damage or oxidative stress. For the GO analysis: Biological Process (BP) terms were predominantly linked to xenobiotic substance metabolism and stress response, lipid metabolism and inflammatory regulation. Molecular Function (MF) terms were mainly associated with oxidation-reduction and detoxification functions, monooxygenase and lipid metabolism, and heme binding and oxygen metabolism. Cellular Component (CC) terms were enriched in processes related to cornified envelope formation, neural synapses, and regulation of cell polarity. (**Figure 2C**).

**Figure 3:**
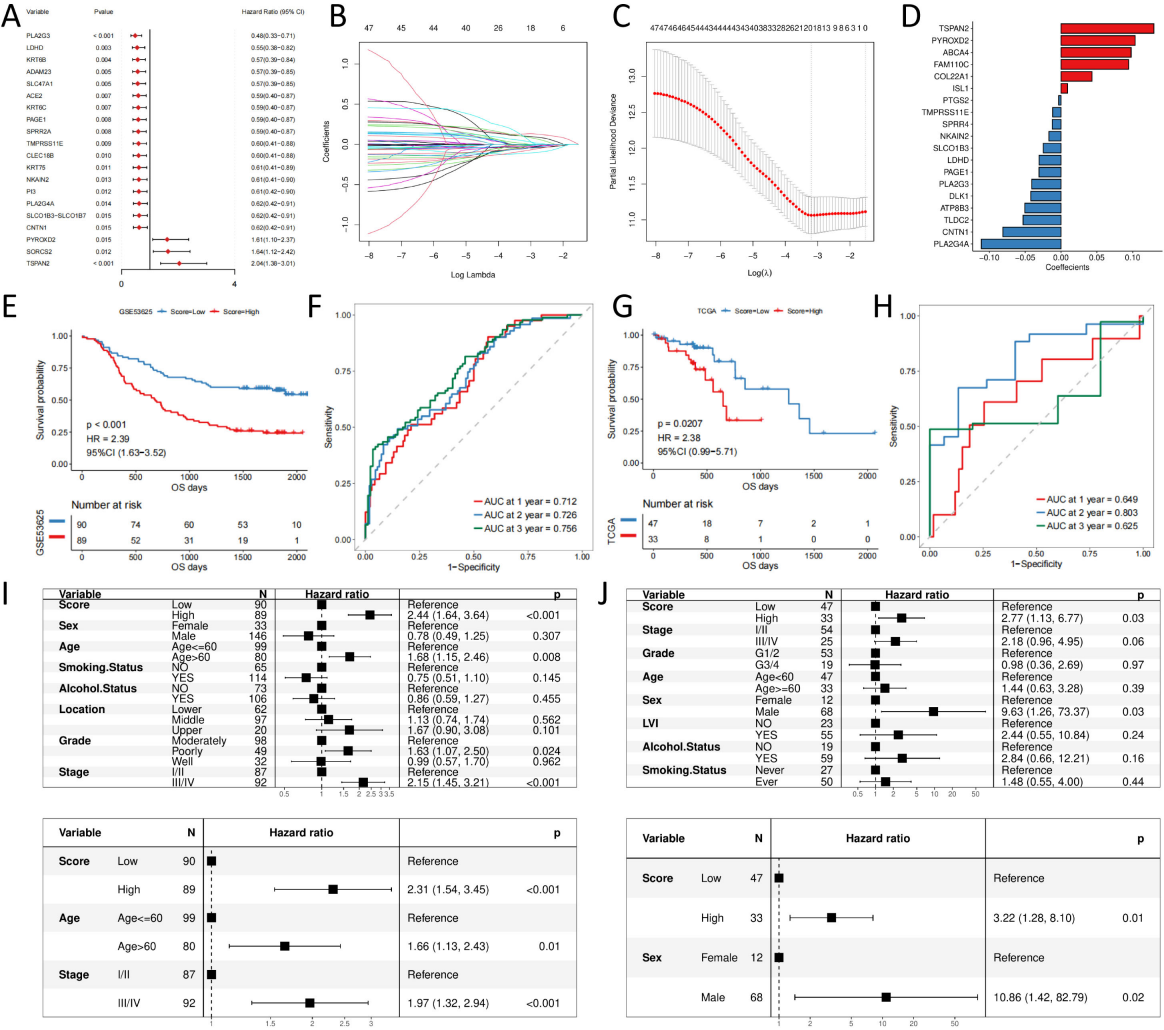
signature construction and validation **(A)** forest plot of prognostic efficacy of top20 prognostic genes; **(B)** confidence interval of each Lambda of LASSO regression; **(C)** change trajectory of LASSO regression independent variables, the horizontal coordinate indicates the logarithm of the independent variable Lambda, and the vertical coordinate indicates the coefficient of the independent variable; **(D)** LASSO regression coefficient of key prognostic genes; **(E)** Prognostic survival curves for high- and low- score groups in the training set; **(F)** ROC curves in the training set; **(G)** Prognostic survival curves for high- and low- score groups in the validation set; **(H)** ROC curves in the validation set; **(I)** Independent prognosis for high and low groups of risk scores in the training set; **(J)** Independent prognosis for high and low groups of risk scores in the validation set.

Next, the univariate Cox regression analysis exposed that 47 of these differential genes were significantly associated with patient OS in the GSE53625 esophageal squamous cancer cohort (**Supplementary Table 1**), and the forest plot visualizes the Cox regression analysis results of the 20 genes with the smallest p-values (**Figure 3A**). **Supplementary Figure 1** displays the KM curves of the six genes with the smallest to the greatest P-values.

Furthermore, LASSO-Cox regression analysis was performed based on these 47 genes. We performed 10-fold cross-validation under optimal conditions to determine the model’s penalty parameter (λ), eliminating 19 key prognostic factors affecting patient OS (**Figures 3B–D**). Based on the key prognostic factor expression levels and the linear combination of the corresponding weights, we construct the signature that can assess each patient’s prognosis. **Table 1** illustrates the coefficients of each factor.

**Table 1:** Key factors and the corresponding coefficients.

signature	coef
*PLA2G3*	-0.0408777770054639
*TSPAN2*	0.129552453050212
*LDHD*	-0.0306569525078317
*PAGE1*	-0.0307708458875278
*TMPRSS11E*	-0.0118258735625092
*NKAIN2*	-0.0169467967600925
*PLA2G4A*	-0.111288936388811
*PYROXD2*	0.10341543327457
*CNTN1*	-0.0811084517504579
*SPRR4*	-0.012175650239528
*TLDC2*	-0.0530741811661962
*ISL1*	0.00907394814271503
*PTGS2*	-0.00337995214635402
*DLK1*	-0.0422476394195441
*ATP8B3*	-0.0505582772279708
*COL22A1*	0.0431952962889701
*ABCA4*	0.0977266256856891
*FAM110C*	0.0943087131900304
*SLCO1B3*	-0.0248740526803297

We calculate the risk score for each patient in the training set based on the constructed prognostic signature and divide them into high- and low-risk groups based on the median value. KM curve analysis and the log-rank test indicated that high-risk group patients had significantly shorter OS (p-value < 0.05, **Figures 3E**). The AUC for the predicted outcome of the sample was 0.712, 0.726, and 0.756 at one, two, and three years respectively (**Figures 3F**), indicating that the Score can provide a good characterization of sample OS. Then, we explored the independence of prognostic signature in the training set. Univariate and multivariate Cox regression models were constructed based on prognostic signature and clinical characteristics, and the results revealed that prognostic signature was an independent prognostic factor (HR = 2.31, p-value < 0.05, **Figure 3I**).

We used TCGA-ESCC as an independent validation set to assess the reliability of the prognostic signature. Patients were divided into high- and low-risk groups according to the prognostic signature risk score. Patient OS was also significantly lower in the high-risk group than in the low-risk group (**Figure 3G**), with predicted outcome AUCs of 0.649, 0.803, and 0.625 for the validation set samples at one, two, and three years, respectively (**Figure 3H**). Univariate and multivariate Cox regression models were constructed based on prognostic signature and clinical characteristics, and the results were consistent with the test set, again supporting prognostic signature as an independent prognostic factor (HR = 3.22, p-value < 0.05, **Figure 3J**).

### Single-cell transcriptome analysis of prognostic signature

**Figure 4:**
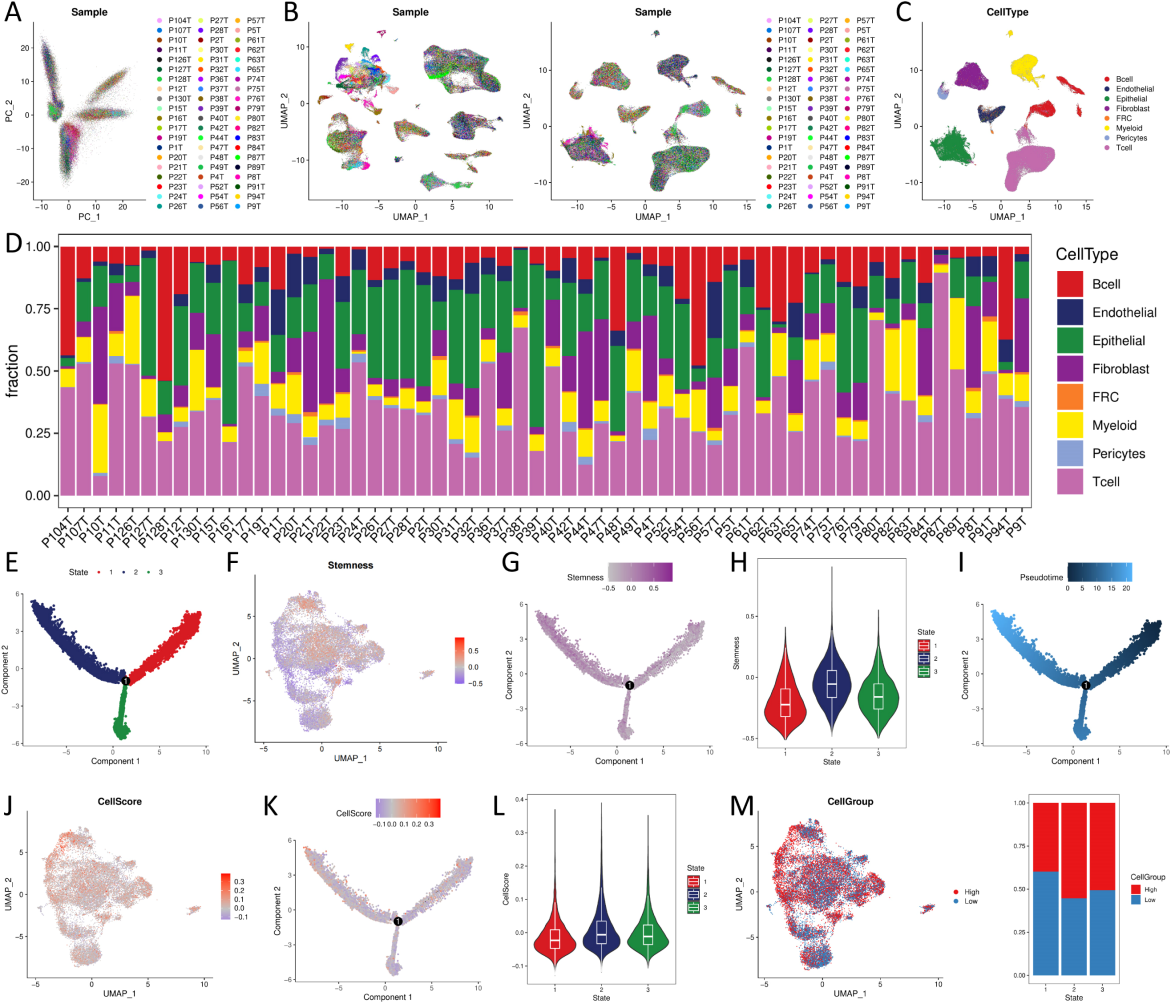
CellScore of single cell sequencing data and trajectory analysis **(A)** PCA analysis; **(B)** cell distribution before and after batch effect; **(C)** cell type distribution; **(D)** cell proportional distribution of samples. **(E, G, I, K)** trajectory distribution of State, Stemness, Pseudotime, CellScore; **(F, J, M)** UMAP plots of Stemness, CellScore, CellGroup of UMAP plot; **(H, L)** violin distribution of Stemness, CellScore in different States; **(N)** proportional distribution of CellGroup in different States.

The original authors of the dataset quality-controlled scRNA-seq data of 192,078 cells (containing 20,335 B cells, 10,346 Endothelial, 44,547 Epithelial, 27,881 Fibroblast, 1,136 Fibroblastic reticular cells (FRC), 18,514 Myeloid, 3,023 Pericytes, and 66,296 T cell). Moreover, 17,986 genes were detected. The PCA results presented a significant batch effect between samples (**Figure 4A**), and the batch effect between samples was removed using Harmony (**Figure 4B**). UMAP demonstrated the distribution of different cell types (**Figure 4C**), and the proportion of cell distribution in each sample was heterogeneous (**Figure 4D**).

We extracted epithelial cells identified by copykat as malignant and with at least one model gene detected (N = 19159, such as at least one model gene with expression greater than 0), calculated Stemness and CellScore for each cell, and classified CellGroup into high and low groups according to CellScore, using UMAP for presentation (**Figures 4F, J, M**). Trajectory analysis of malignant epithelial cells revealed three differentiation states (**Figure 4E**). State1 cells, characterized by low stemness (**Figures 4G, H**), represented the least aggressive phenotype. Tumor progression was associated with a gradual shift from State1 to State2/3, marked by increased stemness and loss of differentiation (**Figure 4I**). State1 → State2 and State1 → State3 trajectory routes have an increased CellScore (**Figures 4K, L**) and an increased proportion of high in the CellGroup (**Figure 4N**), indicating an increase in tumor malignancy.

### Prognostic signature and immune microenvironment

**Figure 5:**
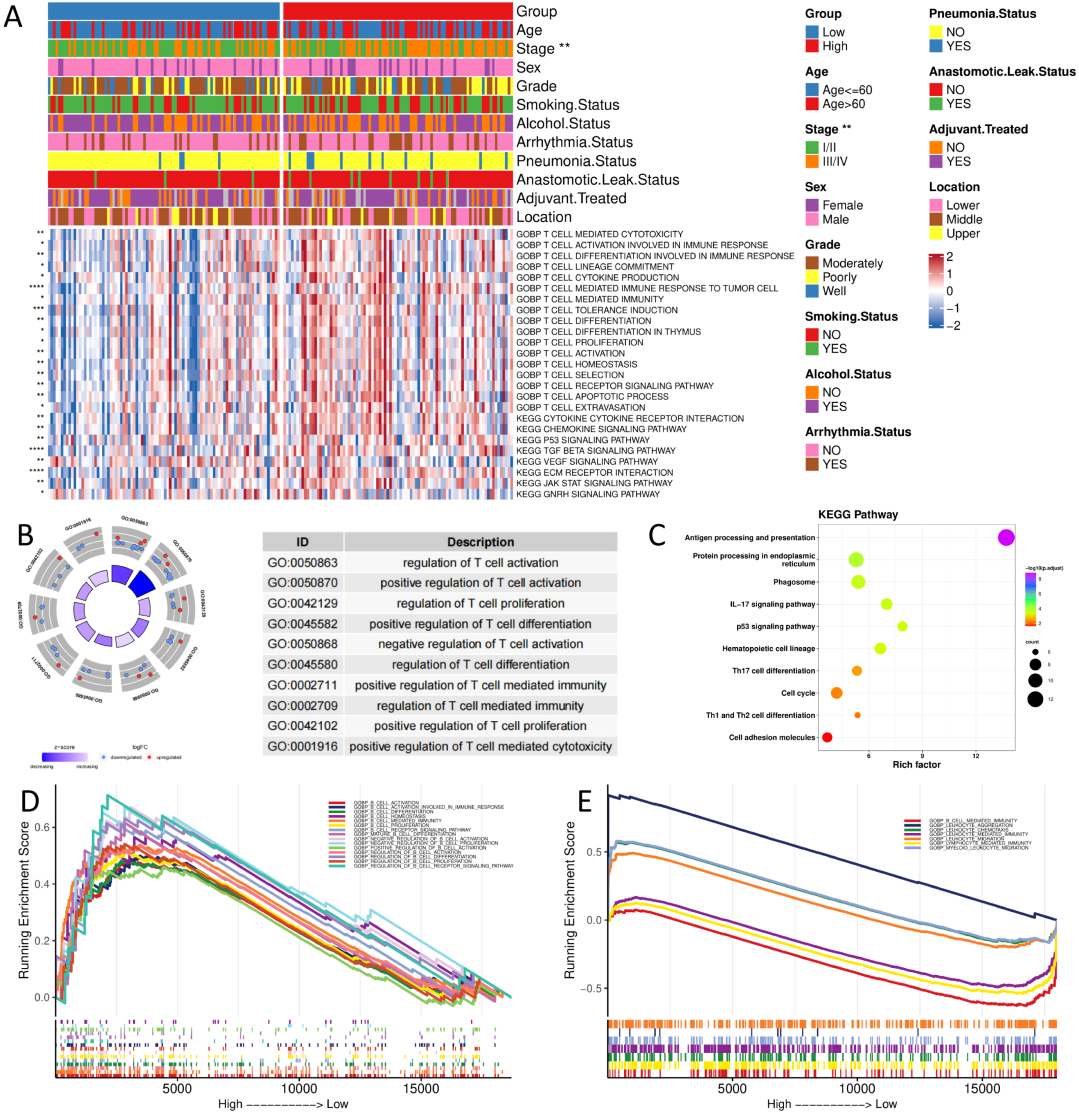
Functional enrichment analysis of different groups **(A)** GOBP and KEGG pathway enrichment analysis of bulk sequencing data; **(B,C)** GOBP and KEGG pathway enrichment analysis of scRNA sequencing data; **(D, E)** GSEA analysis of bulk and scRNA sequencing data. (*, p < 0.05; **, p < 0.01; ***, p < 0.001; ****, p < 0.0001).

Based on the bulk sequencing data, we calculated the pathway/biological process activities of KEGG and GOBP using the GSVA algorithm. We compared the differences between the activities of different groups using the rank sum test. The results demonstrated that the immune-related biological processes activities, such as T cell activation and T cell-mediated tumor cell immune response, were significantly higher in the high-score group than in the low-score group. High-score tumors exhibited stronger activation of pro-tumor pathways, including p53 signaling, chemokine production, and immune cell recruitment (**Figure 5A**).

Based on scRNA-seq data, we calculated differentially characterized genes between high and low groups of CellGroup and performed GO and KEGG enrichment analysis using clusterProfiler. The results presented that genes were significantly enriched in immune-related biological processes, such as regulation of T-cell activation and proliferation, and tumor progression-related pathways, such as cell cycle, P53 signaling pathway, and antigen processing and presentation (**Figures 5B, C**), consistent with the results of bulk sequencing data.

Next, we analyzed the enrichment differences between high and low groups using GSEA. The results disclosed that the high-risk group was significantly enriched in immune-related pathways, such as B-cell activation and lymphocytes, in both bulk and scRNA-seq data (**Figures 5D, E**).

**Figure 6:**
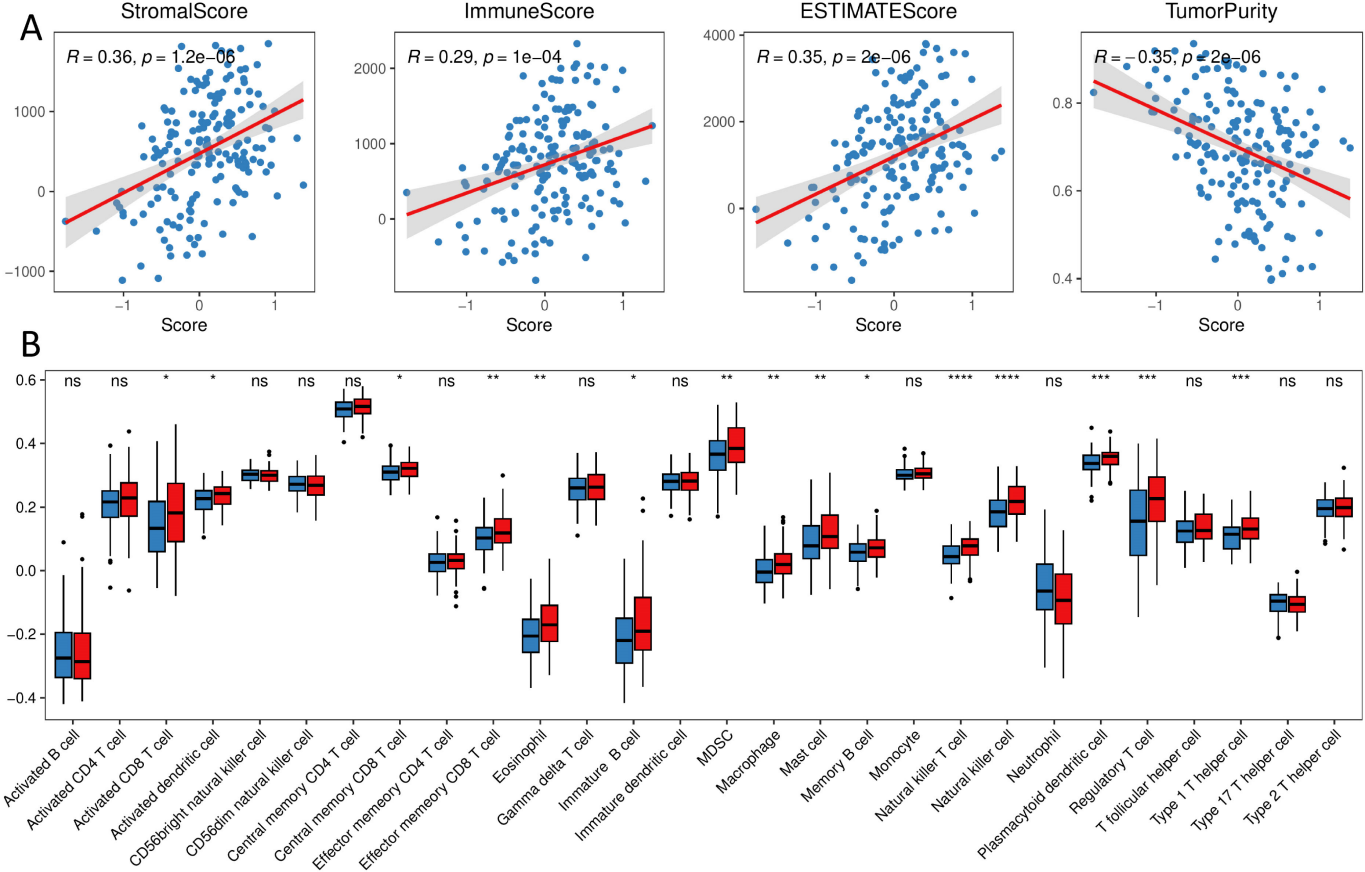
Immune landscape of different groups **(A)** correlation of immune stroma score and tumor purity with the score of prognostic signature; **(B)** distribution of the proportion of immune cell infiltration in different groups. (ns, p > 0.05; *, p < 0.05; **, p < 0.01; ***, p < 0.001; ****, p < 0.0001).

We calculated the percentage of immune cell infiltration in each tumor sample using the bulk sequencing data. The results revealed that the score of prognostic signature was significantly positively correlated with a stromal score, immune score, and ESTIMATE score, and significantly negatively correlated with tumor purity (**Figure 6A**). The percentage of infiltrated cells, such as Natural killer cells, Type1 T helper cells, and regulatory T cells (Tregs), was significantly lower in the Low group samples than in the High group (**Figure 6B**).

### Differences in specific cellular communication between high-and low-prognostic signature score groups

**Figure 7:**
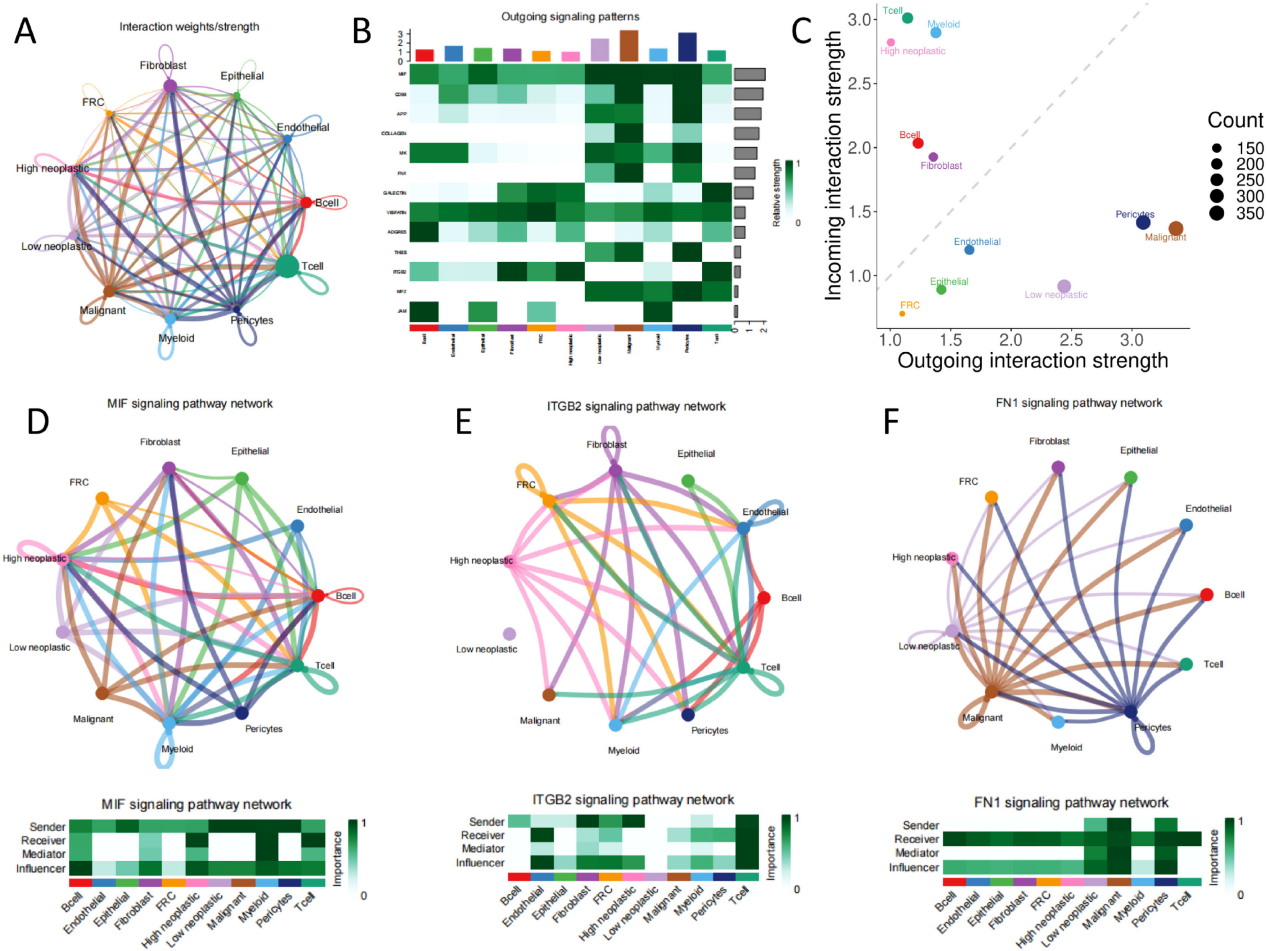
Cellular communication **(A)** all network diagram; **(B)** signaling dominant statistical heat plot; **(C)** signaling dominant statistical point diagram; **(D, E, F)** network of signaling pathways.

Next, we performed intercellular communication analysis using the CellChat package, with extensive cellular communication among the cell populations (**Figures 7A, B**). When distinguishing between incoming and outgoing signaling, Malignant Epithelial Cells, Non-Malignant Epithelial Cells, Pericytes, Low neoplastic, Endothelial, and FRC are outgoing signaling dominant senders, while B cell, T cell, Fibroblast, High neoplastic, and Myeloid are incoming signaling dominant receivers (**Figure 7C**). Low and high neoplastic are in multiple tumor malignant progression-related pathways such as MIF, ITGB2, and FN1 with different cells for messaging (**Figures 7D–F**).

### Potential treatment strategies for prognostic signature

**Figure 8:**
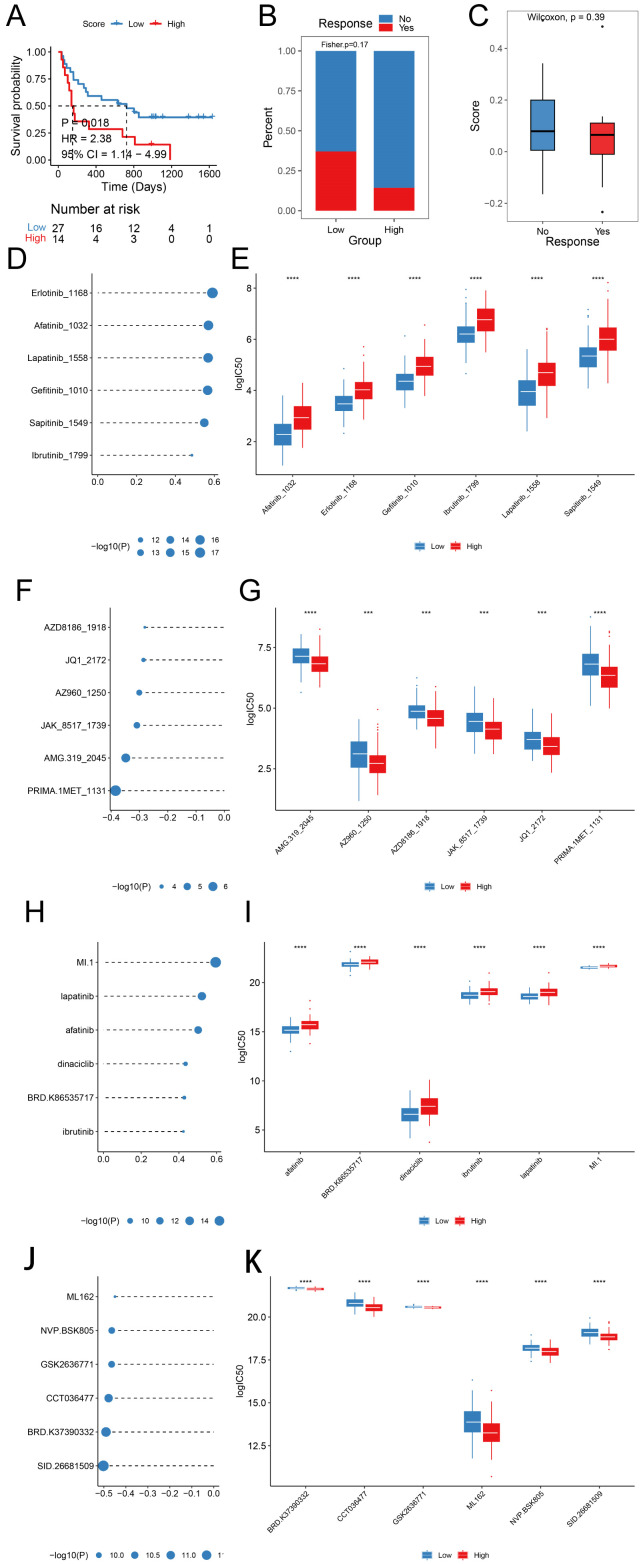
Prognostic signature predicts drug sensitivity **(A)** KM survival curves in different score groups; **(B)** Score distribution in different response groups; **(C)** Distribution of immunotherapy response in different score groups; **(D, E)** Sensitivity analysis of Top 6 drugs with positive correlation with prognostic scores in the GDSC database; **(F, G)** Sensitivity analysis of Top 6 drugs with negative correlation with prognostic scores in the GDSC database; **(H, I)** Sensitivity analysis of Top 6 drugs with positive correlation with prognostic scores in the CTRP database; **(J, K)** Sensitivity analysis of Top 6 drugs with negative correlation with prognostic scores in the CTRP database. (HR, Hazard Ratio; CI, Confidence Interval; ***, p < 0.001; ****, p < 0.0001).

We explored the predictive efficacy of prognostic signature on sample prognosis in the immunotherapy cohort (14). We discovered that patients with the same low score in the immunotherapy cohort had a better prognosis (**Figure 8A**). However, there was no significant difference in risk scores among patients in the immunotherapy with/without response group (**Figure 8B**), nor was there a significant difference in the proportion of samples responding to immunotherapy with/without immunotherapy in the different risk groups (**Figure 8C**).

We predicted the IC_50_ values of the drugs in the training set sample using the R package oncoPredict, GDSC, and CTRP database drug information combined with the expression profile of the training set. We compared the spearman correlation between the score and the IC_50_ of each drug, ranked the drugs according to the absolute value of the correlation coefficient from the largest to the smallest, and selected the top six drugs with significant positive and negative correlations, respectively (**Figures 8D, F, H, J**, p < 0.05), with significant differences in drug IC_50_ across Score groupings (**Figures 8E, G, I, K**).

## Discussion

Recent studies have demonstrated that cells with high SLC7A11 expression exhibit increased susceptibility to cell death under glucose deprivation. This vulnerability primarily stems from reduced glycolysis under glucose-deficient conditions, leading to insufficient NADPH production. Without adequate NADPH, cystine cannot be reduced to cysteine, resulting in disulfide accumulation and subsequent disulfide stress. The accumulated disulfides interact with the actin cytoskeleton, where cross-linking between actin and cytoskeletal proteins disrupts actin architecture, ultimately triggering cell death (15, 16). Termed “disulfidptosis,” this process has been progressively recognized as a form of metabolic cell death, joining ferroptosis and cuprotosis as novel paradigms targeting tumor metabolic vulnerabilities (17–20). While these findings establish fundamental mechanisms of disulfidptosis in solid tumors, its regulatory networks and clinical translation potential in specific malignancies such as ESCC remain underexplored. In this study, we analyzed disulfidptosis-related gene expression differences in ESCC samples from public databases. Unsupervised clustering analysis revealed that higher risk scores were significantly associated with poorer prognosis. To investigate prognostic disparities between high- and low-risk patients, we performed differential gene expression analysis, followed by enrichment analysis, immune infiltration profiling, and drug sensitivithe mechanistic framework of disulfidptosisy evaluation of identified differentially expressed gsurvival curves for prognostic analysis were generated using the Kaplan-Meier methodenes.

ESCC is highly heterogeneous, leading to different clinical outcomes and treatment sensitivity (21). To address this issue, we enriched and analyzed the differential genes between the expression patterns of disulfidptosis and wanted to develop a signature to enable risk stratification and personalized treatment prediction. The findings suggest that tumor progression-related pathways such as DNA adducts, ROS, and lipid metabolism may contribute to prognostic differences in ESCC. The rapid proliferation and metabolic dysregulation of ESCC cells lead to excessive ROS generation, posing a significant threat to cellular survival. To counteract this damage, cells produce glutathione to scavenge excess ROS (22, 23). Furthermore, ROS can oxidize DNA to form adducts, activate DNA damage repair mechanisms, deplete NAD+ reserves, and exacerbate energy crises, indirectly promoting disulfide accumulation (24, 25). Cytochrome P450 enzyme-mediated metabolism of procarcinogens generates DNA adducts while potentially exacerbating reductive stress through NADPH consumption, thereby accelerating disulfide accumulation (26, 27). These signaling pathways are closely associated with the mechanism of disulfidptosis, also indirectly demonstrating that disulfidptosis is a form of cell death induced by disulfide accumulation resulting from cellular metabolic abnormalities.

Tumor microenvironment heterogeneity represents a critical factor in ESCC chemoradiotherapy resistance (18). Our study identified significantly enhanced activity of immune-related BP and tumor progression signaling pathways in the high-risk group, along with substantial differences in immune cell infiltration proportions, including natural killer cells, Th1 cells, and Tregs. Notably, IFN-γ secreted by CD8+ T cells was found to suppress the expression of disulfidptosis-related genes SLC3A2 and SLC7A11—two subunits of the cystine transport system (28, 29). This suggests that the anti-tumor effects of disulfidptosis may involve immunomodulatory mechanisms, potentially through CD8+ T cell functional inhibition or SLC3A2/SLC7A11-mediated cystine metabolism regulation impacting immune escape in ESCC. Importantly, analysis of immunotherapy cohorts confirmed enhanced treatment responsiveness in low-risk patients, indicating the prognostic model’s utility in identifying potential immunotherapy beneficiaries. However, the lack of significant correlation between risk scores and objective response rates to immune checkpoint inhibitors implies that this signature primarily reflects global TME characteristics (e.g., immunosuppressive status or metabolic stress levels) rather than directly influencing antigen presentation efficiency. This observation aligns with features of SLC7A11-high tumors, where previous studies have shown that SLC7A11 overexpression enhances tumor antioxidant capacity through GSH synthesis, fostering an immunosuppressive microenvironment (30, 31).

Targeted therapy, as a new therapeutic method, plays an important role in the treatment of ESCC (32), such as targeting the metabolic vulnerability of SLC7A11-high cancer cells, glucose transporter type 1 biosynthesis, and glutathione induces NADPH dissipation, significant disulfide molecules accumulation, such as cysteine, and ROS accumulation, thereby inhibiting tumor growth and spread (33, 34). Using the oncoPredict algorithm, we identified significantly correlated IC_50_ values for therapeutic agents. Notably, AZD8186 and JQ1 exhibited lower IC_50_ values and stronger cytotoxic effects in high-score tumors, suggesting synergistic interactions between their targeted pathways (PI3K, BET) and score-characterized metabolic vulnerabilities (35, 36). Conversely, high-score ESCC cells demonstrated significant resistance to EGFR inhibitors (e.g., Gefitinib) and BTK inhibitors (e.g., Ibrutinib), potentially mediated through compensatory EGFR pathway activation or drug efflux pump upregulation. While previous studies in bladder, lung, and breast cancers have validated associations between disulfidptosis-related models and chemotherapeutic sensitivity (12, 13, 37), this work establishes the first disulfidptosis-associated drug prediction framework for ESCC, providing a basis for personalized therapeutic strategies.

While mechanistic understanding of disulfideptosis continues to advance, the disease-specific regulatory networks in ESCC remain to be fully elucidated. Real-time dynamic monitoring of metabolic biomarkers including glutathione and ROS is crucial for addressing adaptive therapeutic resistance. Furthermore, the observed dissociation between our risk stratification model and immunotherapy outcomes implies the existence of additional regulatory factors modulating the disulfideptosis-immune crosstalk, which necessitates multi-omics integration for accurate predictive modeling. Finally, computationally identified drug candidates such as AZD8186 and JQ1 require rigorous experimental validation to confirm their therapeutic potential. Future investigations should incorporate systematic approaches combining *in vivo* and *in vitro* experiments to decipher the precise regulatory architecture of disulfideptosis in ESCC. Prospective clinical trials should be implemented to systematically evaluate the therapeutic efficacy of targeted strategies such as SLC7A11/GLUT1 inhibitors, while concurrently advancing the development of enhanced multi-omics-integrated prognostic frameworks.

## Conclusions

This study provides a new prognostic signature based on disulfidptosis for ESCC, which can be used as an effective tool for predicting prognosis. We analyzed the prognostic indicators of ESCC and explored different tumor growth patterns, immune microenvironments, cell communication, and drug therapy, providing new theoretical support for clinical decision-making.

## Data Availability

The datasets presented in this study can be found in online repositories. The names of the repository/repositories and accession number(s) can be found in the article/**Supplementary Material**.
