# Supplementary figures and images for: Identification of disulfidptosis in esophageal squamous cell carcinoma based on single-cell and bulk RNA-seq data to predict prognosis and treatment response
Source: Front Immunol. 2025 Apr 15;16:1567793. doi: 10.3389/fimmu.2025.1567793 (PMC12037556; doi:10.3389/fimmu.2025.1567793)

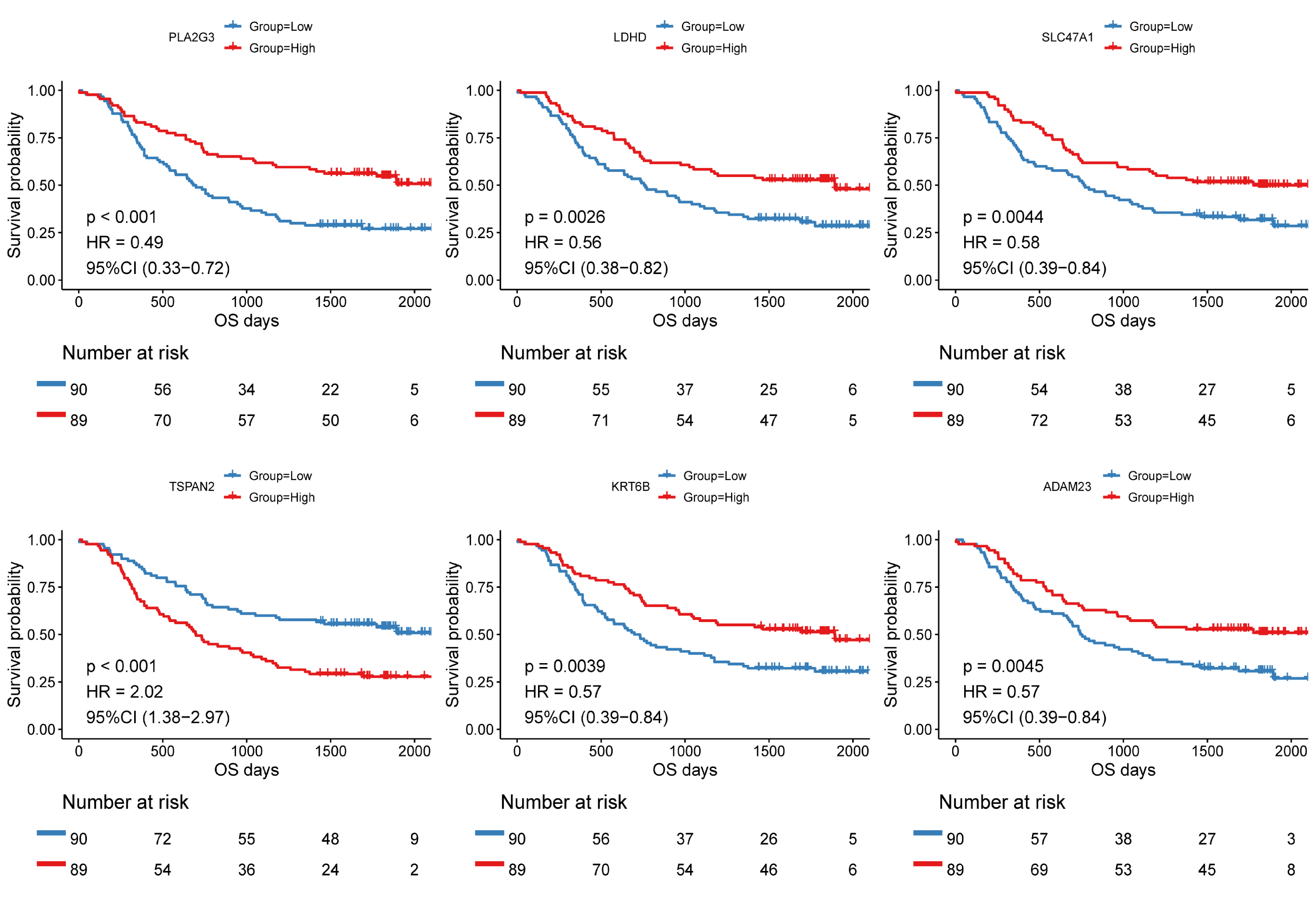

Supplement: Supplementary file 2 [file Image1.tif]
